# The Gene Ontology knowledgebase in 2026

**DOI:** 10.1093/nar/gkaf1292

**Published:** 2025-12-18

**Authors:** The Gene Ontology Consortium, Suzi A Aleksander, James P Balhoff, Seth Carbon, J Michael Cherry, Dustin Ebert, Marc Feuermann, Pascale Gaudet, Nomi L Harris, David P Hill, Patrick Kalita, Raymond Lee, Huaiyu Mi, Sierra Moxon, Christopher J Mungall, Anushya Muruganujan, Tremayne Mushayahama, Paul W Sternberg, Paul D Thomas, Kimberly Van Auken, Edith D Wong, Valerie Wood, Jolene Ramsey, Deborah A Siegele, Rex L Chisholm, Robert Dodson, Petra Fey, Maria Cristina Aspromonte, Maria Victoria Nugnes, Ximena Aixa Castro Naser, Silvio C E Tosatto, Michelle Giglio, Suvarna Nadendla, Giulia Antonazzo, Helen Attrill, Nicholas H Brown, Gil dos Santos, Steven Marygold, Katja Röper, Victor Strelets, Christopher J Tabone, Jim Thurmond, Pinglei Zhou, Rossana Zaru, Ruth C Lovering, Colin Logie, Daiqing Chen, Alexandra Naba, Karen Christie, Lori Corbani, Li Ni, Dmitry Sitnikov, Cynthia Smith, James Seager, Laurel Cooper, Justin Elser, Pankaj Jaiswal, Parul Gupta, Sushma Naithani, Pascal Carme, Kim Rutherford, Jeffrey L De Pons, Melinda R Dwinell, G Thomas Hayman, Mary L Kaldunski, Anne E Kwitek, Stanley J F Laulederkind, Marek A Tutaj, Mahima Vedi, Shur-Jen Wang, Peter D’Eustachio, Lucila Aimo, Kristian Axelsen, Alan Bridge, Nevila Hyka-Nouspikel, Anne Morgat, Gene Goldbold, Stacia R Engel, Stuart R Miyasato, Robert S Nash, Gavin Sherlock, Shuai Weng, Erika Bakker, Tanya Z Berardini, Leonore Reiser, Andrea Auchincloss, Ghislaine Argoud-Puy, Marie-Claude Blatter, Emmanuel Boutet, Lionel Breuza, Cristina Casals-Casas, Elisabeth Coudert, Anne Estreicher, Maria Livia Famiglietti, Arnaud Gos, Nadine Gruaz-Gumowski, Chantal Hulo, Florence Jungo, Philippe Le Mercier, Damien Lieberherr, Patrick Masson, Ivo Pedruzzi, Lucille Pourcel, Sylvain Poux, Catherine Rivoire, Shyamala Sundaram, Alex Bateman, Aduragbemi Adesina, Emily Bowler-Barnett, David Carpentier, Paul Denny, Alexandr Ignatchenko, Rizwan Ishtiaq, Antonia Lock, Yvonne Lussi, Michele Magrane, Maria J Martin, Sandra Orchard, Pedro Raposo, Elena Speretta, Nidhi Tyagi, Nadya Urakova, Kate Warner, Conny Wing-Hen Yu, Juancarlos Chan, Stavros Diamantakis, Mark Quinton-Tulloch, Daniela Raciti, Malcolm Fisher, Christina James-Zorn, Virgilio Ponferrada, Aaron Zorn, Doug Howe, Sridhar Ramachandran, Leyla Ruzicka, Monte Westerfield

## Abstract

The Gene Ontology (GO) knowledgebase (https://geneontology.org) is a comprehensive resource describing the functions of genes. The GO knowledgebase is regularly updated and improved. We describe here the major updates that have been made in the past 3 years. The ontology and annotations have been expanded and revised, particularly in several areas of biology: cellular metabolism, multi-organism interactions (e.g. host-pathogen), extracellular matrix proteins, chromatin remodeling (e.g. the “histone code”), and noncoding RNA functions. We have released version 2 of a comprehensive set of integrated, reviewed annotations for human genes, which we call the “functionome.” We have also dramatically increased the number of GO-CAM models, with over 1500 models of metabolic and signaling pathways, primarily in human, mouse, budding and fission yeast, and fruit fly. Finally, we discuss our current recommendations and future prospects of AI in the use and development of GO.

## Graphical Abstract

**Graphical Abstract:**
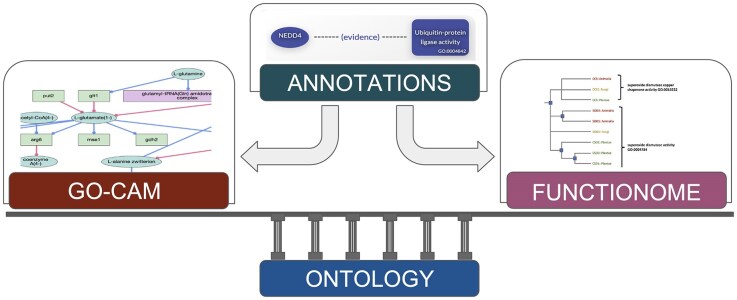

## Introduction

Genes encode gene products, often proteins but also non-coding RNA molecules (ncRNAs), that perform functions at the molecular, cellular, and organismal levels. The GO knowledgebase provides a comprehensive, structured, computer-accessible representation of gene functions for genes from any cellular organism or virus. The GO knowledgebase defines the functions of genes, using three types of functional characteristics: molecular function (MF), cellular component (CC), and biological process (BP) [1, 2], as shown in Table 1.

**Table 1.** The three aspects of function represented in the Gene Ontology

Ontology aspect	Short label	Definition	Examples
Molecular function	MF	A molecular process that can be carried out by the action of a single macromolecular machine, usually via direct physical interactions with other molecular entities. Function in this sense denotes an action, or activity, that a gene product (or a complex) performs.	*DNA-binding transcription factor activity* (GO:0003700) *molecular adaptor activity* (GO:0060090)
Cellular component	CC	A location, relative to cellular compartments and structures, occupied by a macromolecular machine. There are three types of cellular components described in the gene ontology: (i) the cellular anatomical entity, where a gene product carries out a molecular function; (ii) virion components, where viral proteins act; and (iii) the stable macromolecular complexes of which gene products are parts.	*plasma membrane* (GO:0005886) *mitochondrion* (GO:0005739 *virion nucleoid* (GO:0039642) *clathrin complex* (GO:0071439)
Biological process	BP	The execution of a genetically encoded biological module or program. It consists of all the steps required to achieve the specific biological objective of the module. A biological process is accomplished by a particular set of molecular functions carried out by specific gene products (or macromolecular complexes), often in a highly regulated manner and in a particular temporal sequence.	*DNA-templated DNA replication* (GO:0006261) *tricarboxylic acid cycle* (GO:0006099)

In short, a gene product, located in a *cellular component*, performs a *molecular function* as part of a *biological process*. A biological process generally involves multiple gene products with distinct molecular functions. Conversely, a given gene product may perform multiple molecular functions in different cellular components and/or as part of different biological processes.

The Gene Ontology (“GO ontology”) is a knowledge structure describing the types of molecular functions, cellular components, and biological processes that are currently known. The GO ontology consists of GO terms (e.g. the molecular function term *DNA-binding transcription factor activity*, with the unique identifier GO:0003700) and relations between GO terms (e.g. *DNA-binding transcription factor activity* is_a *transcription regulator activity*, where the relation is_a means “is a subtype of”). The BP and CC hierarchies also include part_of relationships, allowing a pathway to be broken down into sub-steps, or an organelle into sub-components. A GO annotation is an association between a specific gene product (i.e. the active product of a gene) and a GO term, and should be interpreted as a statement that the specified gene product performs the specified functional characteristic represented by the GO term. Each GO annotation is based on evidence, which can be broadly characterized as direct experimental evidence or indirect evidence of different types (https://geneontology.org/docs/guide-go-evidence-codes/). Each GO annotation covers only a single characteristic of gene function, and multiple GO annotations are generally required to completely describe the function of a gene product. GO Causal Activity Models (GO-CAMs) combine multiple GO annotations into models of biological processes or “pathways” that show how different gene products interact in larger systems [3].

The GO serves a wide variety of use cases. These range from serving as an encyclopedic knowledgebase allowing for reliable and trusted lookup of functional information for a gene in human or any other species, to providing the primary tool for analysis of complex omics data. The GO is the standard tool for enrichment analysis and gene set analysis, allowing biologists to obtain mechanistic insights from gene expression data and multiple other kinds of omics data [4]. GO is incorporated in tools that perform single-cell analysis, such as Seurat [5]. In addition to being a fundamental tool for molecular biology and clinical research, it is increasingly deployed for clinically relevant tasks such as disease gene prioritization [6]. More recently, the GO has been used as a source of training and verification for multiple different artificial intelligence (AI) models [7].

## Updates to the GO knowledgebase contents

The components of the GO knowledgebase are updated continuously to extend, enhance, and refine the ontology and the annotations. The GO knowledgebase is packaged and released regularly. The most current version of the data can be found at https://current.geneontology.org/. The GO ontology is available in different editions, as described in Table 2. All changes to the ontology and the annotations are tracked at each release, and this information is reported on our website at https://geneontology.org/stats.html.

**Table 2.** GO ontology editions

GO edition	Format(s)	Relations included	Links to other ontologies	Number of relations
go-basic	OBO, JSON, and OWL-RDF/XML	is_a, part_of, regulates, negatively_regulates, and positively_regulates	Not supported	39 906
go	OBO, JSON, and OWL-RDF/XML	Same as go-basic, plus has_part, and occurs_in	Not supported	78 889
go-plus	JSON and OWL-RDF/XML	Same as go, plus 65 other relation types (download at https://geneontology.org/docs/download-ontology/)	ChEBI, Uberon, Cell Ontology, Sequence Ontology, CARO, Dicty Anatomy, Fungal Anatomy Ontology, Plant Ontology, PATO, Protein Ontology	121 698

Editions are distinguished by the relations and metadata they include. All editions are updated at each GO release. External ontologies used in GO include: ChEBI [8], Uberon [9], Relation Ontology [10], Cell Ontology [11], Sequence Ontology [12], Dicty Anatomy [13], CARO [14], Fungal Anatomy Ontology (https://obofoundry.org/ontology/fao), Plant Ontology [15], PATO [16], and Protein Ontology [17].

Changes (Table 2) in GO contents may affect GO enrichment analysis results [18, 19] and other analyses that rely on the GO knowledgebase. In order to ensure reproducibility, whenever publishing analyses performed on or using the GO ontology, annotations, and/or GO-CAM models, users should always cite the release version. For example, all the statistics and descriptions reported in this article compare the GO release 2025-07-22 (https://release.geneontology.org/2025-07-22/ doi:10.5281/zenodo.16423886) with that of 2025-11-03 (https://release.geneontology.org/2022-11-03; doi:10.5281/zenodo.7407024), described in the previous update [20]. Archived versions of GO can be found on the GO website (https://release.geneontology.org/) and at the Open Research Repository Zenodo (https://zenodo.org/records/17382285).

### Updates to the ontology

Table 3 shows the number of terms currently in the ontology, as well as the numbers of GO terms added and removed (obsoleted; with or without a direct replacement term) over the past 3-year period, for each aspect of GO. Substantial changes have been made to the ontology since our last update, especially in the biological process and molecular function aspects of the ontology. In total, 768 new terms were added (an increase of ∼2% in the total number of terms), while 4173 terms were removed (a decrease of ∼10% in the total number). Note that GO has stopped merging terms since 2022; instead, terms are obsoleted and replaced by other terms using the “replaced_by” OBO tag. The ontology has undergone substantial revision and improvement, with over 4000 terms added or removed. Many of the added terms reflect improvements to the ontology in specific areas of biology (see the “updates to the GO in specific domains of biology” section below). The removed terms represent ongoing efforts to simplify, remove redundancy, and improve modularization of the ontology and to make the BP branch of GO consistent with the definition of a BP as a “a genetically encoded biological module or program” [2]. Broad categories for term obsoletions are shown in Table 4. Users can expect GO-based analyses to be affected to a greater degree for the types of GO terms, and specific areas of biology, that have been the focus of the most extensive changes. 

**Table 3.** Current status and changes to GO terms in the past three year period

GO aspect	Total number of terms	Added terms	Obsoleted terms
Molecular function	10 155	359	1483
Cellular component	4052	103	90
Biological process	25 699	306	2600
Total	39 906	768	4173

**Table 4.** Common reasons for term obsoletion with corresponding examples

Reason for obsoletion	Examples
Phenotype of perturbation experiments	*negative regulation of necrotic cell death* (GO:0060547)
BP terms that represent a MF	*histone H2A acetylation* (GO:0043968)
Pre-composed terms that are now represented by GO-CAMs	*histone H4 acetylation involved in response to DNA damage stimulus* (GO:2000776)
Sub-reaction and reaction mechanisms	*formation of peptidyl-cystine persulfide by sulphur transfer from free cysteine* (GO:0044526)
Substrates beyond the specificity of known enzymes or not physiologically relevant	*4,4′-diapophytoene desaturase (4,4′-diapolycopene-forming)* (GO:0140868)

### Updates to gene sets

GO annotations are also updated due to updates in the set of genes that are currently available for each organism, e.g. when there is a new genome assembly for a given organism or when the gene structure annotations are changed. Generally, these sets become more accurate over time. Automatic annotation pipelines are run at each release and manual annotations are reviewed for potential updates whenever there is a large change in the sequence of the gene product.

### Creating and revising GO annotations

GO annotations derived from experimental data are added by the annotation groups in the GO Consortium (https://geneontology.org/docs/whoweare/). There are also several computational pipelines that produce annotations using more indirect evidence, many of which are carefully reviewed by experts to ensure accuracy, e.g. InterPro2GO [21] or rule-based approaches, such as UniRule [22]. The sources and pipelines for GO annotations have not been changed since our last update paper [20], and we refer readers to this paper as well as online documentation (https://geneontology.org/docs/go-annotations) for more information.

New GO annotations are continually added to the knowledgebase to reflect the latest experimental findings and to fill in annotation gaps using older, but previously uncurated, publications. In addition, GO annotations are regularly reviewed and may be updated or removed from the knowledgebase when ontology terms are revised (see the “Updates to the GO in specific areas of biology” section below), when annotations are invalidated by new experimental evidence, or when annotation guidelines are revised. When ontology terms are slated for obsoletion, annotations to those terms are manually reviewed and replaced with a different ontology term whenever possible. For example, the obsoletion of *methylated histone binding, acetylated histone binding*, and related terms led to the manual review of over 2000 annotations. These binding terms did not sufficiently capture the biological relevance of the binding, which was addressed by the addition of histone writer/reader/eraser activities, which specify whether the histone is a substrate (for histone writers and erasers) or an adaptor (for readers).

In the past three years, experimentally supported, new manually curated annotations have been extracted from roughly 15 000 peer-reviewed scientific publications. As of July 2025, the GO knowledgebase contains 1 135 364 experimentally supported annotations from over 185 000 peer-reviewed publications (this excludes direct annotations to *protein binding*, GO:0005515), compared to 996 903 in the 11 November 2022 GO release, a 12% net increase, i.e. the difference between new annotations added and annotations that were removed.

## The human functionome: a new, comprehensive resource of reviewed GO annotations

The Phylogenetic Annotation using GO (PAN-GO) approach of GO annotation has been in place for over 15 years [23]. This approach integrates experimental annotations from multiple organisms, using an explicit model of gene function evolution. For each protein family tree, the model specifies branches along which functions, represented as GO terms, were gained or lost, taking into account events such as gene duplications, sequence divergence, and horizontal gene transfers, as well as taxonomic specificity. These annotations are identified by the “IBA” (inferred from biological ancestor) evidence code and cover the 144 organisms included in PANTHER gene families (http://pantherdb.org/panther/summaryStats.jsp) [24].

**Figure 1.**
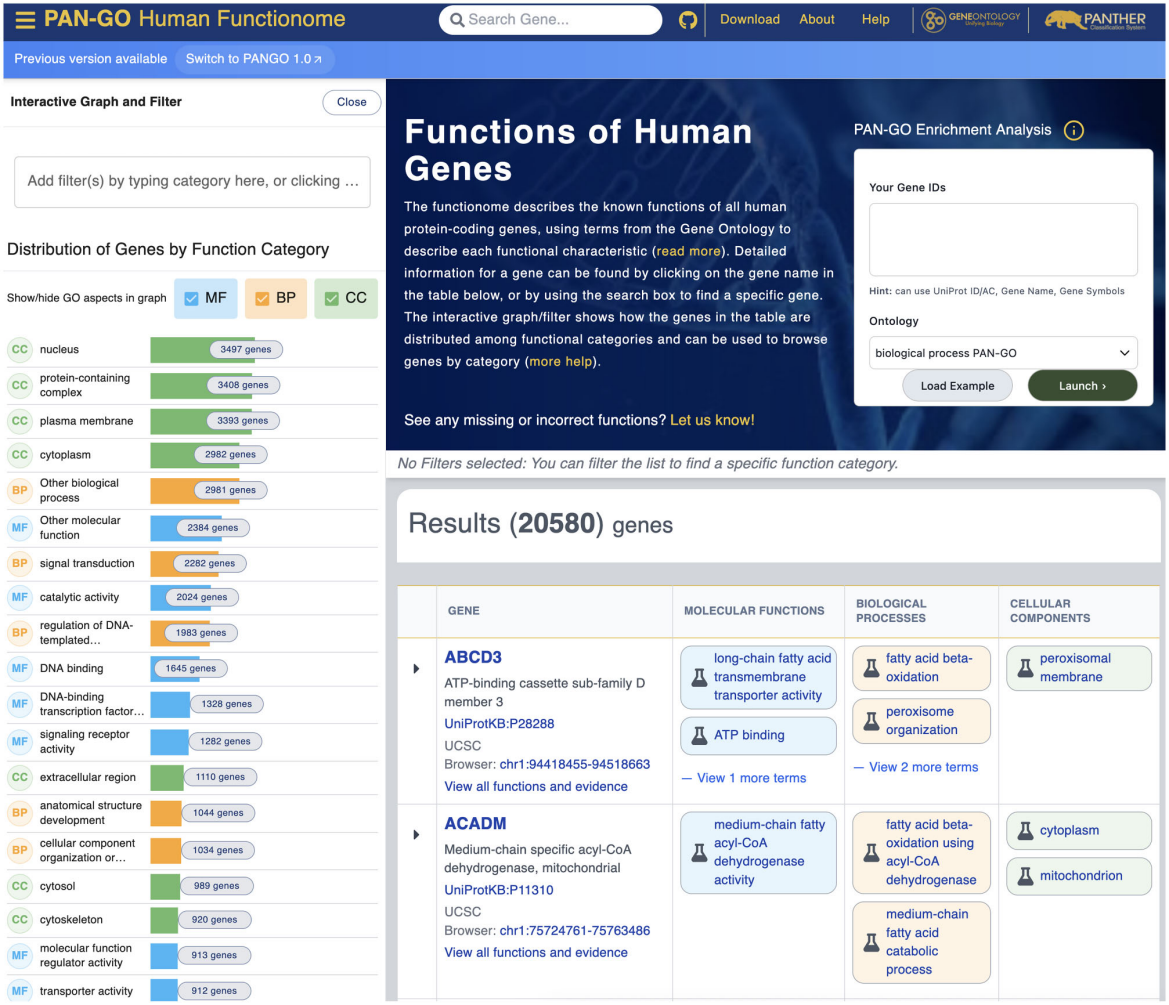
PAN-GO human functionome website.

The initial phase of the PAN-GO project focused on comprehensive coverage of human genes, by modeling function evolution of all gene families that contain at least one human gene. This phase was recently completed, and the resulting set of comprehensive, reviewed GO annotations for human genes (the human gene “functionome”) are described in detail elsewhere [25]. To facilitate access and use of this resource, we have created a dedicated website at https://functionome.geneontology.org (Fig. 1). The PAN-GO annotations covered significantly more protein-coding genes than experimental evidence alone: 82% of human genes versus 68%. We have shown that enrichment analysis using only this set of annotations, compared to using the full set of all human GO annotations, dramatically reduces the number of enriched GO terms while still identifying the biologically relevant terms [25]. Enrichment analysis with human PAN-GO (IBA) annotations can be launched directly from the PAN-GO Human Functionome site (https://functionome.geneontology.org/).

We report here the release of version 2.0 of the PAN-GO functionome, which will be further updated regularly. Human protein-coding gene coverage has increased to 84% of human genes (an increase of 2% compared to version 1.0) and 47% of genes have GO annotations in all three aspects of MF, BP, and CC (an increase of 5% compared to version 1.0). Gene annotations have been reviewed and improved for thousands of human genes. Version 2.0 is shown by default at https://functionome.geneontology.org, with a link to the older version so users can compare them in detail.

### Functionomes for other genomes

PAN-GO models utilize phylogenetic trees covering genes from 144 organisms across the tree of life. As a result, functionomes can be constructed in principle for not only the human genome, but also other genomes as well. Due to the experimental GO annotations currently available, the PAN-GO annotation coverage of vertebrate genomes is substantially higher than that of other genomes (Table 5). Coverage depends on the progress of the PAN-GO annotation of families, as well as the availability of experimental GO annotations, as all PAN-GO annotations are based on experimental evidence for at least one related gene. As of July 2025, a total of 11 036 protein families (out of 11 952 families with available experimental GO annotations) have been curated at least once. In addition, the PAN-GO evolutionary models are improved as new experimental annotations become available, and from feedback from GO Consortium member groups and GO users. Feedback forms are available on the human functionome website, or users can just contact the GO Helpdesk.

**Table 5.** Genome coverage of PAN-GO annotations

Taxonomic group	Gene coverage of annotations (mean ± SD)
vertebrates	78% ± 5%
invertebrates	49% ± 11%
fungi	54% ± 15%
plants	43% ± 6%
protists, alveolates, amoebae	41% ± 9%
archaea	35% ± 4%
bacteria	49% ± 12%

Percentage of protein-coding genes with at least one PAN-GO reviewed annotation for different taxonomic groups.

Currently, there is no website for browsing non-human functionomes, and these GO annotation sets must be downloaded. For the 144 genomes in the phylogenetic trees annotated by PAN-GO, the annotations are available in all the standard GO annotation download files at http://geneontology.org and can be identified by filtering for the “IBA” evidence code. For a large number of other genomes, PAN-GO annotations have been generated computationally via the TreeGrafter tool [26] available in InterProScan [27]. UniProt has applied this pipeline to all the UniProt Proteomes, which can be browsed using the QuickGO tool [28]. NCBI has applied the pipeline to all of the eukaryotic genomes in RefSeq, and GO annotation files can be downloaded (https://geneontology.org/docs/download-go-annotations). These annotations are considered computationally inferred (IEA evidence code) and can be identified by the contents of the “Reference” field of the annotation files: reference GO_REF:0000118 (https://geneontology.org/GO_REF/0000118) in QuickGO, or PMID:30032202 in NCBI RefSeq files.

## GO causal activity models (GO-CAMs)

**Figure 2.**
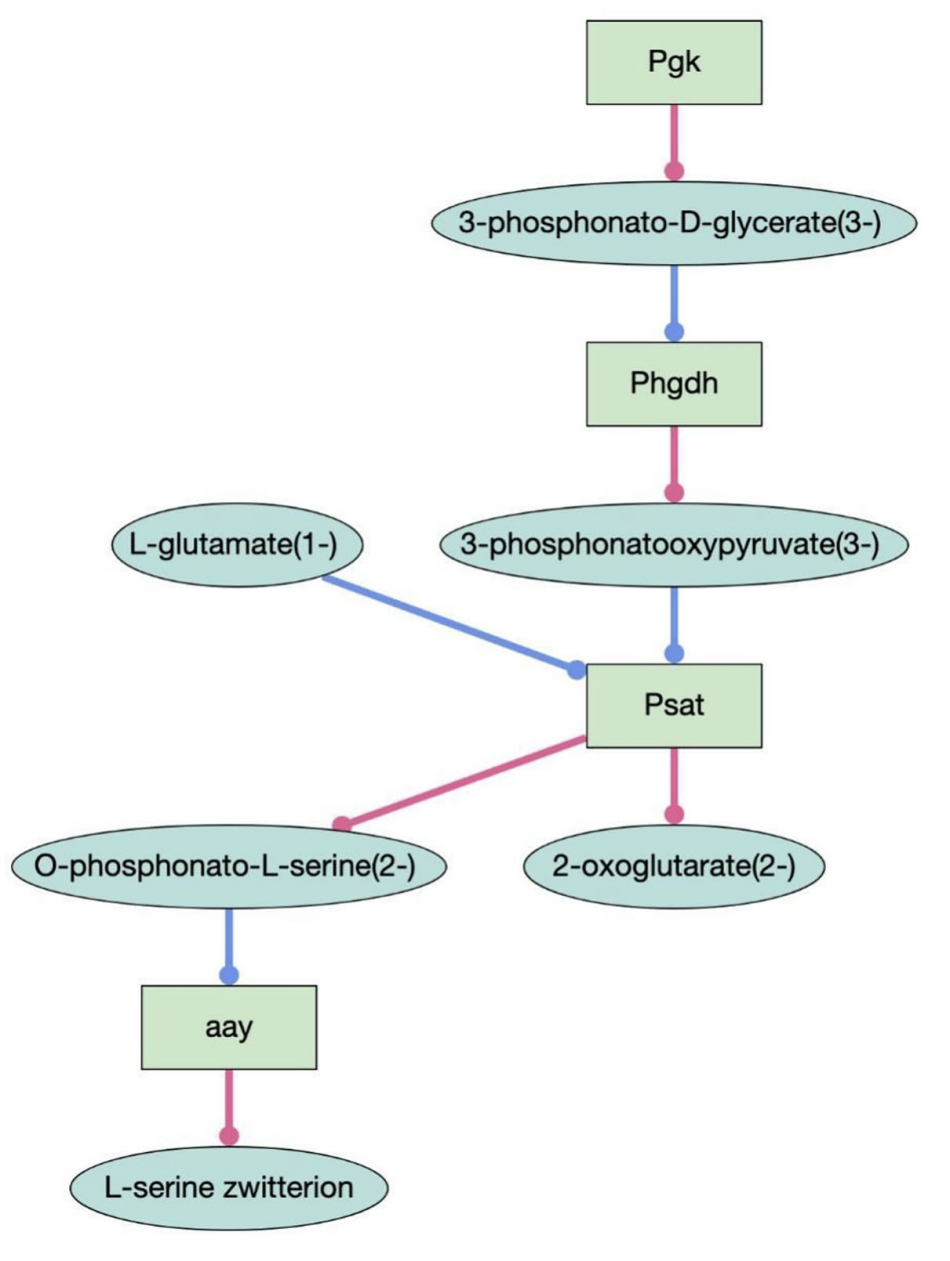
GO-CAM model of serine biosynthesis in *D. melanogaster* as displayed using the GO-CAM Pathway Widget (code available at https://github.com/geneontology/web-components). For simplicity, gene product activities (rectangles) are labeled only with the gene that encodes the activity, and not the additional information in the GO-CAM about the linked GO annotations and evidence (these can be accessed by clicking). Small molecule substrates (blue lines) and products (pink lines) of enzymatic activities are shown in ovals, and are taken from the ChEBI ontology.

GO-CAMs are models of causal “pathways” that describe how different gene products work together in a biological system [3]. A GO-CAM links the activities (GO molecular functions) of gene products together by causal relations that specify the effect of one activity on the other [3, 20]. An example is shown in Fig. 2. GO-CAMs are an extension of the GO annotation model, and like GO annotations, each element of a GO-CAM is supported by evidence (see Thomas *et al*. [3] for details). GO-CAMs correspond to activity flow representations, which are complementary to the process diagram representations in Reactome. There are many differences in these representations [3], but one practical difference is that GO-CAMs tend to be simpler in structure, as they capture a causal flow (including positive and negative effects) between individual gene product activities, while Reactome models capture a sequence of physical binding and catalysis events that involves a much larger number of physical entities and molecular complexes. We continue to make progress on a longer-term project to align the Reactome resource and GO-CAMs [29], which will result in Reactome-derived GO-CAM models, but these are not yet publicly available and will be described in a future publication.

In the past 3 years, the number of expert-curated GO-CAM pathways has increased nearly five-fold, with 1571 pathways available as of July 2025. Models are constructed by expert, trained curators, using the Noctua Visual Pathway Editor software, using a standardized training process and documentation. The list of pathways can be found at https://geneontology.org/go-cam. This GO-CAM website has been updated to improve usability and response time. Table 6 lists the number of GO-CAMs available, as well as the number of gene products included in these models, for the organisms with the highest coverage. GO-CAMs are accessible from the GO website homepage, by clicking on the “Browse GO-CAMs” link. GO-CAMs can be viewed as pathway diagrams (Fig. 2) or retrieved via API (https://api.geneontology.org/) or downloads (https://geneontology.org/docs/download-go-cams/). All GO-CAMs can be viewed and downloaded on the GO website or on the relevant gene pages at the Alliance of Genome Resources [30] (https://alliancegenome.org). GO-CAMs for human genes are also available on the relevant UniProt [31] (https://uniprot.org) protein pages, and GO-CAMs for *S. pombe* are available at PomBase. The human GO-CAMs are also available through the Network Data Exchange (NDEx) [32] site at https://home.ndexbio.org/index/, where users can use the iQuery tool to search GO-CAMs and find GO-CAMs that are enriched for genes in a user-specified gene list.

**Table 6.** Expert-curated GO-CAM pathway models available as of July 2025

Organism	Number of GO-CAM models	Number of distinct gene products in pathways
Human	907	1606
Mouse	275	845
Budding yeast *(S. cerevisiae*)	197	533
Fission yeast (*S. pombe*)	81	873
Fruit fly (*D. melanogaster*)	67	388

GO-CAMs provide more than a biologically intuitive representation of molecular processes; they establish a causal framework that enables powerful interpretation of experimental data and systematic interrogation of established biological knowledge to generate new hypotheses. As a proof of principle, PomBase has constructed an integrated “mega model” that allows users to overlay gene lists onto GO-CAM representations. This integration supports the analysis of experimental results and the identification of unexpected patterns or inconsistencies that merit further investigation. Through this approach, several biological anomalies have already been identified, including non-essential genes within apparently essential linear pathways, which highlight potential loci for further genetic analysis. Moreover, numerous “missing proteins” have been revealed, representing cases in which molecular activities are known but the corresponding entities remain unidentified or ambiguous. Mapping disease classes or phenotypic traits onto the mega model can further uncover novel candidate genes underlying specific biological processes or pathogenic mechanisms. As these models continue to mature, it will become increasingly feasible to trace the downstream effects of mutations in individual genes, thereby advancing our capacity to model, predict, and interpret complex biological systems.

## Updates to the GO in specific domains of biology

The next sections describe specific areas of the ontology that have undergone major refactoring since our last update in 2023. Annotations have also been revised to reflect these changes.

### Updates to enzymatic functions

We have made a large effort to align the GO *catalytic activity* (GO:0003824) branch with Enzyme Commission (EC) [33] and Rhea [34]. Rhea uses the ChEBI ontology of chemical entities [8] to represent chemical entities in a standardized, consistent manner. Over 2000 cross-references to these resources were updated: mappings to obsolete Rhea and EC entries were either updated or the term was obsoleted; ambiguous Rhea/EC/MetaCyc/KEGG [35, 36] mappings were resolved; redundant and out-of-scope GO terms were obsoleted, resulting in over 1000 terms obsoleted in that branch. This includes terms that represent enzyme substrate descriptions that were too specific, as well as non-physiological chemical substrates. Finally, we have added automated QC checks for mappings to make sure that mappings to external resources resolve to a valid identifier. There are now over 9400 RHEA and EC terms mapped to GO terms.

### Metabolic process refactoring

We have started a major refactoring of the *metabolic process* (GO:0008152) branch of the biological process aspect of the ontology. One area of focus is the conflation, in previous versions of GO, of *metabolic processes* with any cellular chemical conversion. We have narrowed the definitions and usage of GO terms to ensure that chemical conversions are represented as molecular functions, while biochemical pathways leading to the construction of cellular molecules and macromolecules or their degradation to simple products are defined as metabolic processes (biosynthesis and catabolism). Hence, conversion of one molecule to another is not necessarily a biological process in itself. For example, *DNA methylation on cytosin*e (GO:0032776) was obsoleted because it was redundant with the molecular function *DNA (cytosine-5-)-methyltransferase activity* GO:0003886. Many of the genes previously annotated to the obsolete terms were reannotated instead to the more informative biological process *DNA methylation-dependent constitutive heterochromatin formation* (GO:0006346).

### Multi-organism interactions

We have extensively revised biological process terms that describe multi-organism interactions, with a particular focus on supporting the description of microbial pathogenesis processes. We have simplified and aligned three aspects of host/pathogen interactions, such that we now have three main branches under *modulation of process of another organism* (GO:0035821): *host-mediated perturbation of symbiont process* (GO:0051851), *symbiont-mediated perturbation of host process* (GO:0044003), and *venom-mediated perturbation of biological process* (GO:0035738). Part of this work was recently published [37].

These ontology terms and their relationships are useful for pathway modeling, as well as for querying annotations to satisfy recent US Government requirements for screening sequences that endow a microbe with pathogenic function for human hosts (https://aspr.hhs.gov/S3/Documents/OSTP-Nucleic-Acid-Synthesis-Screening-Framework-Sep2024.pdf). The new and revised biological process terms focus on the role of relevant microbial genes in subverting host processes, particularly innate immunity.

### Extracellular matrix

**Figure 3.**
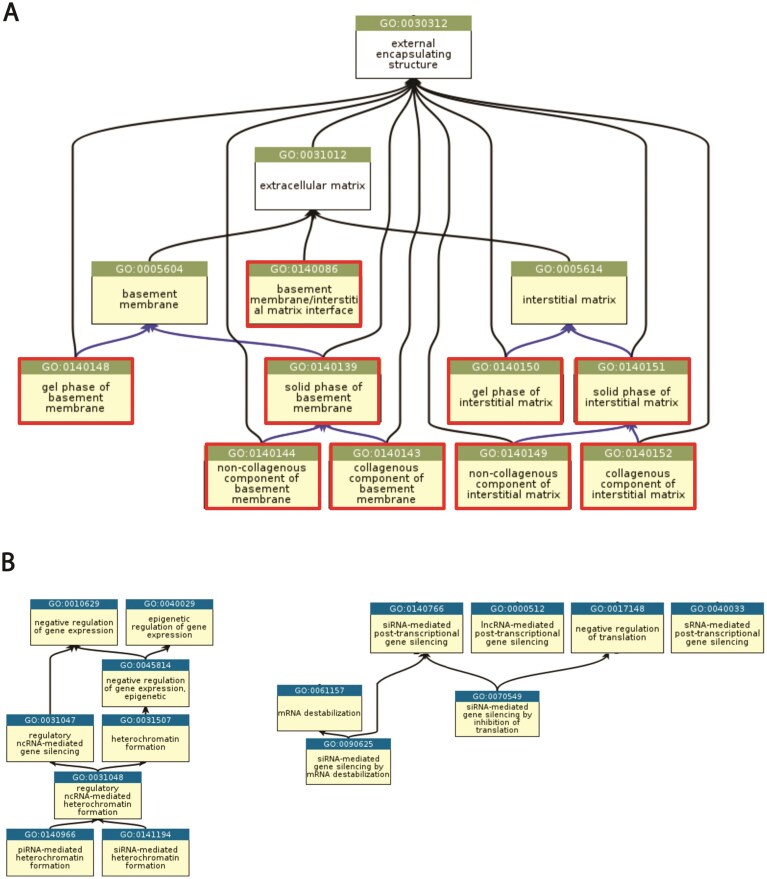
Graphical representation highlighting ontology changes. (**A**) the extracellular matrix cellular branch of the cellular component, in which newly created terms are indicated by a red box, and (**B**) a selection of processes mediated by non-coding RNAs. Black lines represent is_a relations, while blue lines represent part_of relations.

We have initiated a collaboration with the Matrisome Project (https://matrisome.org) [38, 39] on the revision of the terms pertaining to the extracellular matrix (ECM). The ECM is a complex scaffold of proteins and glycans, often viewed as the glue that holds cells together and that organizes cells into tissues and tissues into organs [40, 41]. The ECM is found in all multicellular organisms and governs or modulates most cellular processes, including cell proliferation, adhesion, migration, or differentiation [42, 43, 44]. The “matrisome,” the ensemble of genes encoding structural and regulatory components of the ECM, constitutes ∼4% of the mammalian genome [45]. We have updated and refined the representation of ECM structures where molecular functions are performed. We have added a term representing the interface connecting the basement membrane and the interstitial matrix, the *basement membrane/interstitial interface* (GO: 0140086), and created terms to more precisely describe the ECM substructures (Fig. 3A). We also reviewed and updated all terms and annotations pertaining specifically to collagens, the most abundant proteins in vertebrates. Finally, we have created a new grouping term for *specialized ECM* (GO:0140047) and completed the list of these structures. This work is ongoing, and we will next curate and update MF and BP terms associated with ECM gene products. While our initial efforts are primarily directed toward mammalian ECMs and gene products, the ECM is present in some form in all multicellular organisms. We and others have built lists of the matrisomes of multiple model organisms [46]. It will thus be important to harmonize all new GO terms pertaining to the ECM across all organisms.

### Epigenetics, chromatin remodeling & transcription

We have reviewed and reorganized the branch representing the epigenetic processes that occur during multicellular organism development: *epigenetic programming of gene expression* (GO:0043045), *genomic imprinting* (GO:0071514), and s*ex-chromosome dosage compensation* (GO:0007549), to make sure that the terms and their ontological organization are consistent with the specific epigenetic processes that occur in different organisms for gametogenesis and following fertilization.

Another epigenetic aspect we addressed is to create novel histone writer, reader, and eraser activities, replacing modified histone binding terms that were much less specific. There are now over 200 GO ontology terms describing histone modifying and reader activities, such as the histone writer *histone H3K36 methyltransferase activity* (GO:0046975), the reader *histone H3K36me3 reader activity* (GO:0140003), and the eraser *histone H3K36 demethylase activity* (GO:0051864). Similarly, molecular functions were created to describe the modifications that take place on the heptad repeats residues (YSPTSTS) of carboxy-terminal domain of the largest RNA polymerase subunit. The heptad repeat is present in over 50 copies, and each of the residues is subject to modification, either proline isomerization or (de)phosphorylation of serine, threonine, and tyrosine. These modifications are central to the transcription cycle, from transcription initiation to RNA polymerase recycling. These improvements to the molecular functions branch of GO now support causal modeling of epigenetic signaling and chromatin remodeling processes such as *transcription initiation-coupled chromatin remodeling* (GO:0045815), *transcription elongation-coupled chromatin remodeling* (GO:0140673), *heterochromatin formation* (GO:0031507), *DNA replication-dependent chromatin assembly* (GO:0006335) and *disassembly* (GO:0140889), *DNA repair-dependent chromatin remodeling* (GO:0140861), as well as *CENP-A containing chromatin assembly* (GO:0034080) for chromosome segregation.

### Non-coding RNA

To improve the annotation of the regulatory classes of ncRNAs, namely microRNA (miRNA), PIWI-interacting RNA (piRNA), small interfering RNA (siRNA), and long non-coding RNA (lncRNA), we have conducted an extensive review of the literature, revising the ontology and introducing new terms to more precisely describe the activity of ncRNAs. These are accompanied by guidelines, annotation examples, and annotation decision trees to ensure curation consistency (recently described in Antonazzo *et al*. 2024 [47]). From this review, we concluded that the small (∼20–35 nt) regulatory ncRNAs—miRNA, siRNA, and piRNA—fit into discrete models of action [48, 49] that could be captured using standardized GO term patterns. The GO knowledgebase now contains over 108 000 annotations to ncRNAs of various types. Most of these are for ncRNA genes in selected mammals (human, pig, dog, rat, cow, mouse), but many are also available for the plant *A. thaliana*, invertebrates *D. melanogaster* and *C. elegans*, and the fungi *S. cerevisiae* and *S. pombe*. About 13 000 of these are manual annotations supported by direct experimental evidence, another 5000 are manually reviewed based on other evidence, and the remaining 90 000 are computationally inferred. The available annotations can be browsed and downloaded at https://amigo.geneontology.org/amigo/search/annotation.

Regulatory ncRNAs can mediate post-transcriptional gene silencing (PTGS) in the cytosol by base-pairing with mRNA targets. This common mechanistic basis is described by the MF term *mRNA base-pairing post-transcriptional repressor activity* (GO:1903231). Selected biological process terms representing small ncRNA-mediated gene expression regulation via various mechanisms are shown in (Fig. 3B).

LncRNAs present a much more diverse range of activities [50], often more akin to proteins, ranging from organizing nuclear architecture to post-transcriptional regulation. Many lncRNAs act to bring macromolecules together to initiate a process. These functional characteristics are described under the *molecular adaptor activity* (GO:0060090) molecular function branch of the ontology. New terms have been created to describe lncRNA activities, e.g. *dsDNA*–*RNA triple helix-forming chromatin adaptor activity* (GO:0141180), and some are more generally applicable e.g. *molecular condensate scaffold activity* (GO:0140693). We have provided case studies and annotation guidelines [47] and https://wiki.geneontology.org/LncRNA_GO_annotation_manual).

## Ontology subsets

**Figure 4.**
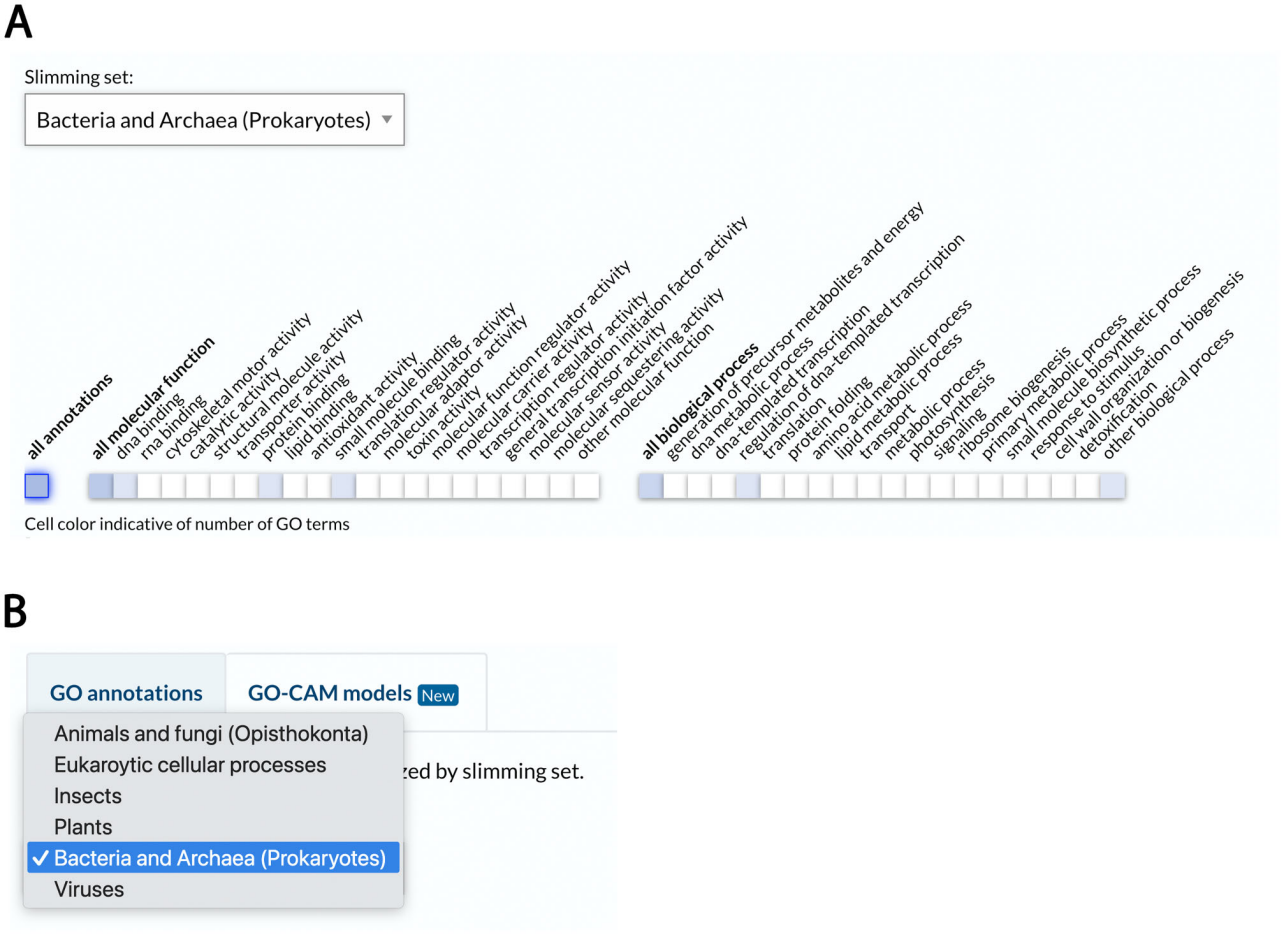
New GO subsets, as viewed in the “GO ribbon” widget on UniProt protein pages. (**A**) An example subset used for an overview of GO annotations for a bacterial gene. (**B**) A drop-down menu for selecting between different subsets.

GO subsets (sometimes known as "GO slims") are condensed versions of the GO ontology containing only a portion of the terms. Subsets are specified by tags within the ontology files (Table 2) that indicate that a given term is a member of a particular subset. GO subsets are particularly useful for providing a global overview of the functions of all the genes in a genome and even an overview of the different GO annotations for a single gene. In the last update we described the updated “GO Generic subset,” a subset that aims to be general and applicable to any species [20]. We have since produced subsets tailored to various taxonomic clades: animals and fungi (opisthokonts), unicellular eukaryotes, insects, plants, prokaryotes, and viruses. For multi-species databases, it is now possible to select and use these different subsets based on the specific organism. Figure 4A shows an example of a UniProt entry for a bacterial gene, with the gene functions summarized by the prokaryotes GO ribbon. A user can use the pull-down menu to select a different subset if desired (Fig. 4B). The GO subsets can be accessed at https://current.geneontology.org/ontology/subsets/ in OWL, JSON, and TSV. We have also developed a metadata file (https://current.geneontology.org/ontology/subsets/go_subsets_metadata.yaml) that allows users to select the appropriate subset for their use.

## Accessing and downloading GO data

### Browsing the ontology and annotations

GO and associated annotations can be searched directly from the Gene Ontology home page (https://geneontology.org/), queried using the AmiGO browser (https://amigo.geneontology.org), or the QuickGO tool (https://www.ebi.ac.uk/QuickGO/) [28]. Gene set enrichment analysis is also directly accessible from the Gene Ontology home page, which runs the PANTHER gene analysis tool at http://pantherdb.org/webservices/go/overrep.jsp [51]. The Human PAN-GO functionome can be browsed and downloaded at https://functionome.geneontology.org/.

### Ontology and annotation downloads

The most current version of the knowledgebase contents can be found at https://current.geneontology.org/. The GO ontology is available in different editions, as described in Table 2. These editions are also described on the GO downloads page https://geneontology.org/docs/download-ontology/. Releases of the ontology and annotations are archived at https://release.geneontology.org/ and on Zenodo (https://doi.org/10.5281/zenodo.1205166). Detailed instructions for downloading GO annotations can be found in the GO documentation: https://geneontology.org/docs/download-go-annotations/. GO-CAM models can be browsed and downloaded at https://geneontology.org/go-cam.

### Documentation

Extensive user documentation can be found on the GO website at https://geneontology.org/. Curation guidelines are available on the GO wiki: https://wiki.geneontology.org/Annotation_guidelines. The data and processes used to produce the GO data are also described in the GO wiki: https://wiki.geneontology.org/Release_Pipeline.

## Discussion

We have described here the major changes to the GO knowledgebase over the past 3 years. The focus has been in three broad areas: improving the representation of biological functions in the GO ontology, increasing the coverage (of published function determination via experiments) and quality of gene function annotations, and increasing the number and biological scope of GO-CAM models. In the ontology we have added over 750 new GO terms, mostly to reflect changes in specific areas of biology in collaboration with experts in these areas. This has also resulted in obsoletion of imprecise or outdated GO terms. In addition, thousands of GO terms have been obsoleted due to top-down refactoring of the ontology, along with review of the annotations to these terms. For many years, the ontology grew organically in a bottom-up fashion, in response to requests from curators who were annotating specific experimental findings and could not identify an appropriate existing GO term. This process continues, but at a decreasing rate, as in most areas of biology the ontology is reasonably complete. For the last 5 years, we have devoted more resources to top-down review of existing ontology terms and annotations to those terms, and remove (i.e. obsolete) terms that unnecessarily complicated the structure of the ontology, had few or inconsistent annotations (often because the term was imprecise), or were redundant with other ontology terms. This review was also performed for entire branches of the ontology, in collaboration with experts in the relevant domain. From user feedback, we find that one of the most common user concerns is the large number of similar GO terms that are often identified in GO enrichment analysis. Many of the improvements reported here, including simplification of the ontology and identification of the “core” gene functions in the human gene “functionome” (https://functionome.geneontology.org) help to reduce the noise of extraneous enrichment results. We plan to continue this work to constantly improve the GO knowledgebase.

Another major advance in the past three years is the number and coverage of GO-CAMs for several organisms. GO-CAMs represent the newest product in the GO knowledgebase and have several advantages over the “standard” GO annotations. First, GO-CAMs link previously independent GO annotations together into a larger model of biological pathways and processes. The GO-CAM evidence model allows multiple pieces of evidence to be used in a single assertion, which may allow for better assessment of confidence in individual statements of the model. In addition, GO-CAM formalizes the previously loosely defined “molecular biology paradigm” underlying GO [2], which has increased the consistency and accuracy of both standard GO annotations and GO-CAM models. For users, GO-CAM allows pathway visualization, as well as analyses using new tools like network-based and GO-CAM model-based enrichment, in addition to the traditional GO term-based enrichment. We expect to see the number of new GO-CAM models continue to grow in each release of the knowledgebase and will continue to improve access to these models via API and website improvements.

### The gene ontology in the age of AI

The advent of artificial intelligence (AI) brings new opportunities both for using GO in research and for assisting with gene function prediction, curation, and ontology development. One of the key transformative developments in AI has been the development of the Transformer architecture (https://arxiv.org/abs/1706.03762), which underpins both text-based large language models (LLMs) exemplified by ChatGPT, as well as genome and protein language models [52] (gLMs and pLMs).

Text LLMs are highly adept at tasks such as summarization and text extraction, so naturally the biocuration community has been exploring the use of these for a variety of tasks [53, 54]. The GO and a number of other ontologies participated in an evaluation of the use of LLMs for ontology term creation task [55]. While this showed some promise, rigorous evaluation with multiple curators showed that the AI would give plausible yet subtly incorrect term definitions for some terms, and that this was only noticed by more expert curators. More recently, more powerful reasoning-based models, coupled with large context windows, have allowed the use of newer agentic AI approaches. With these approaches, the AI system is given a complex task and a set of tools with which to perform that task. The AI system plans the task and coordinates the execution of tools, potentially also invoking other more specialized agents. This approach is well aligned with the complex needs of GO ontology development and curation, which frequently involves integrating multiple sources of information and lines of evidence. We have deployed an ontology agent to assist with tasks such as researching and creating terms. The agent combines the use of LLMs for planning and reasoning, together with rigorous tools for validating changes, such as the ROBOT ontology validation tool [56]. We are currently in the process of evaluating the agentic workflow.

In addition to curation tasks, the summarization capabilities of text LLMs provide opportunities to assist with summarization and interpretation of complex datasets. One of the main use cases for the GO is the interpretation of gene sets or differential expression data from high-throughput experiments and, increasingly, single-cell data. A number of groups have applied LLMs to gene set analysis [57, 58]. The key challenge here is the evaluation of results. Currently, we recommend the use of established statistical methods for gene set analysis that provide rigorous use of the ontology and knowledgebase to provide meaningful rankings of GO term descriptors.

Function prediction from sequence is a promising area in which the latest AI technologies, and in particular pLMs, could provide functional annotations for genes comprising the “unknowme,” i.e. those for which we don’t know the function [59]. AI models like AlphaFold2 have been successfully applied to predicting structure from sequence [60] and for predicting biomolecular interactions [61], so a natural next step is to apply this for function prediction. A number of machine learning methods have been published that claim high accuracy of function prediction from sequence, but rigorous curation of these results has shown that these methods still have major room for improvement [62]. The GO Consortium collaborates closely with the CAFA [63] group to evaluate new AI methods for function prediction. The most recent CAFA competition had a record number of participants, many of whom submitted deep learning-based AI tools. However, as yet, these predicted annotations do not reach the level of precision needed for GO annotations. GO strives to be a reliable source of reviewed and curated knowledge, and for this reason, these predictions are not currently included in the GO knowledgebase.

Novel AI architectures also open up new use cases for the GO and opportunities for innovation. These include the use of GO in knowledge-based verification for generative AI reasoning models. The Chan Zuckerberg Initiative’s rBio model uses GO as a “soft verifier” during reinforcement learning training of a conversational biology virtual cell model LLM (https://doi.org/10.1101/2025.08.18.670981). GO provides reward signals to ensure model outputs align with established biological facts, acting as a knowledge-driven constraint layer that grounds the model in real biology. Another example is how inter-ontology links in the GO between molecular function terms and chemical entities are being used to train transformer models for substrate prediction for classes such as transporters [64]. Similarly, the logical axioms in the GO have been used to train advanced neuro-symbolic AI models to improve on function prediction when compared with sequence alone [65].

## Summary

The Gene Ontology (GO) knowledgebase (https://geneontology.org) is a comprehensive resource of gene function in a structured, standardized form that facilitates use in computational methods and analysis. It is updated continually, with new, updated releases multiple times per year. In the past three years, roughly 800 terms have been added to the ontology, and new experimentally supported gene function annotations have been added from over 15 000 publications. In addition, we have added over 1000 manually curated GO-CAM models (representing causal “pathways” and other biological processes). For human protein-coding genes, we have released a comprehensive, integrated set of annotations of human gene functions: the PAN-GO Functionome (https://functionome.geneontology.org).

## Data Availability

All Gene Ontology code and resources are freely available for download and reuse. Software (https://github.com/geneontology) is under the BSD 3-Clause open-source license. Downloads are available under the CC BY 4.0 license from https://geneontology.org/docs/downloads/.

## Appendix: *Gene Ontology Consortium authors

GO Central: Suzi A. Aleksander (ORCID:0000-0001-6787–2901), James P. Balhoff (ORCID:0000-0002-8688–6599), Seth Carbon (ORCID:0000-0001-8244–1536), J. Michael Cherry (ORCID:0000-0001-9163-5180), Dustin Ebert (ORCID 0000-0002-6659-0416), Marc Feuermann (ORCID: 0000-0002-4187-2863), Pascale Gaudet (ORCID:0000-0003-1813-6857), Nomi L. Harris (ORCID:0000-0001-6315-3707), David P. Hill (ORCID 0000-0001-7476-6306), Patrick Kalita (ORCID 0000-0002-6150-307X), Raymond Lee (ORCID 0000-0002-8151-7479), Huaiyu Mi (ORCID:0000-0001-8721-202X), Sierra Moxon (ORCID: 0000-0002-8719-7760), Christopher J. Mungall (ORCID 0000-0002-6601-2165), Anushya Muruganujan (ORCID 0000-0001-7169-5864), Tremayne Mushayahama (ORCID 0000-0002-2874-6934), Paul W. Sternberg (ORCID 0000-0002-7699-0173), Paul D. Thomas (ORCID 0000-0002-9074-3507), Kimberly Van Auken (ORCID 0000-0002-1706-4196), Edith D. Wong (ORCID:0000-0001-9799-5523), Valerie Wood (ORCID 0000-0001-6330-7526)

CACAO & EcoliWiki: Jolene Ramsey (ORCID: 0000-0002-3774-5896), Deborah A. Siegele (ORCID: 0000-0001-8935-0696)

dictyBase: Rex L. Chisholm (ORCID 0000-0002-5638-3990), Robert Dodson (ORCID 0000-0002-2757-5950), Petra Fey (ORCID 0000-0002-4532-2703)

DisProt: Maria Cristina Aspromonte (ORCiD: 0000-0002-4937-6952), Maria Victoria Nugnes (ORCID: 0000-0001-8399-7907), Ximena Aixa Castro Naser (ORCID: 0000-0002-9211-1255), Silvio C.E. Tosatto (ORCiD: 0000-0003-4525-7793)

ECO: Michelle Giglio (ORCID: 0000-0001-7628-5565), Suvarna Nadendla (ORCID: 0000-0003-3643-281X)

FlyBase: Giulia Antonazzo (ORCID 0000-0003-0086-5621), Helen Attrill (ORCID 0000-0003-3212-6364), Nicholas H. Brown (ORCID 0000-0002-8958-7017), Gil dos Santos (ORCID 0000-0003-3507-8273), Steven Marygold (ORCID 0000-0003-2759-266X), Katja Röper (ORCID 0000-0002-3361-766X), Victor Strelets (ORCID 0000-0001-6556-9335), Christopher J. Tabone (ORCID 0000-0001-8746-0680), Jim Thurmond (ORCID 0000-0002-5142-2583), Pinglei Zhou (ORCID 0000-0002-3012-1044), Rossana Zaru (ORCID: 0000-0002-3358-4423)

Functional Gene Annotation, University College London: Ruth C. Lovering (ORCID: 0000-0002-9791-0064)

GREEKC: Colin Logie (ORCID: 0000-0002-8534-6582)

The Matrisome Project: Daiqing Chen (ORCID 0009-0003-5996-5181), Alexandra Naba (ORCID 0000-0002-4796-5614)

MGI: Karen Christie (ORCID 0000-0001-5501-853X), Lori Corbani (ORCID 0000-0002-2366-557X), David P Hill (ORCID 0000-0001-7476-6306), Li Ni (ORCID 0000-0002-9796-7693), Dmitry Sitnikov (ORCID 0000-0003-3394-9805), Cynthia Smith (ORCID 0000-0003-3691-0324)

PHI-base: James Seager (ORCID 0000-0001-7487-610X)

Planteome: Laurel Cooper (ORCID 0000-0002-6379-8932, Justin Elser (ORCID 0000-0003-0921-1982), Pankaj Jaiswal (ORCID 0000-0002-1005-8383)

Plant Reactome: Parul Gupta (ORCID 0000-0002-0190-8753), Pankaj Jaiswal (ORCID 0000-0002-1005-8383), Sushma Naithani (ORCID 0000-0001-7819-4552)

PomBase: Pascal Carme (ORCID 0009-0003-9059-1333), Kim Rutherford (ORCID 0000-0001-6277-726X), Valerie Wood (ORCID 0000-0001-6330-7526)

RGD: Jeffrey L. De Pons, Melinda R. Dwinell (ORCID 0000-0002-9528-3618), G. Thomas Hayman (ORCID 0000-0002-9553-7227), Mary L. Kaldunski (ORCID 0000-0003-3645-6803), Anne E. Kwitek (ORCID 0000-0003-1024-4116), Stanley J. F. Laulederkind (ORCID 0000-0001-5356-4174), Marek A. Tutaj (ORCID 0000-0002-1025-101X), Mahima Vedi (ORCID 0000-0001-5361-6739), Shur-Jen Wang (ORCID 0000-0001-5256-8683)

Reactome: Peter D’Eustachio (ORCID 0000-0002-5494-626X)

Rhea: Lucila Aimo (ORCID: 0000-0003-0943-6401), Kristian Axelsen (ORCID: 0000-0003-3889-2879), Alan Bridge (ORCID: 0000-0003-2148-9135, Nevila Hyka-Nouspikel (ORCID: 0000-0001-7855-209X), Anne Morgat (ORCID: 0000-0002-1216-2969)

SIgnatureScience: Gene Goldbold (ORCID:0000-0002-5702-4690)

SGD: Suzi A. Aleksander (ORCID:0000-0001-6787-2901), J. Michael Cherry (ORCID:0000-0001-9163-5180), Stacia R. Engel (ORCID:0000-0001-5472-917X), Stuart R. Miyasato (ORCID:0000-0001-5250-8920), Robert S. Nash (ORCID:0000-0002-3726-7441), Gavin Sherlock (ORCID:0000-0002-1692-4983), Shuai Weng (ORCID:0000-0003-4233-0772), Edith D. Wong (ORCID:0000-0001-9799-5523)

The Arabidopsis Information Resource (TAIR): Erika Bakker (ORCID: 0000-0001-7933-3817), Tanya Z. Berardini (ORCID: 0000-0002-3837-8864), Leonore Reiser (ORCID: 0000-0003-0073-0858)

UniProt, Swiss-Prot group: Andrea Auchincloss (ORCID: 0000-0002-5297-5390), Kristian Axelsen (ORCID: 0000-0003-3889-2879), Ghislaine Argoud-Puy (ORCID: 0000-0002-2979-8613), Marie-Claude Blatter (ORCID: 0000-0002-7474-1499), Emmanuel Boutet (ORCID: 0000-0002-3743-4203), Lionel Breuza (ORCID: 0000-0002-8075-8625), Alan Bridge (ORCID: 0000-0003-2148-9135), Cristina Casals-Casas (ORCID: 0000-0001-8769-177X), Elisabeth Coudert (ORCID: 0000-0001-8314-404X), Anne Estreicher (ORCID: 0000-0001-6828-2508), Maria Livia Famiglietti (ORCID: 0000-0002-5283-6593), Marc Feuermann (ORCID: 0000-0002-4187-2863), Arnaud Gos (ORCID: 0000-0002-5018-1378), Nadine Gruaz-Gumowski (ORCID: 0000-0002-4699-4907), Chantal Hulo (ORCID: 0000-0001-8176-7999), Nevila Hyka-Nouspikel (ORCID: 0000-0001-7855-209X), Florence Jungo (ORCID: 0000-0002-7456-8390), Philippe Le Mercier (ORCID: 0000-0001-8528-090X), Damien Lieberherr (ORCID: 0000-0002-9724-1710), Patrick Masson (ORCID: 0000-0001-7646-0052), Anne Morgat (ORCID: 0000-0002-1216-2969), Ivo Pedruzzi (ORCID: 0000-0001-8561-7170), Lucille Pourcel (ORCID: 0000-0003-1522-9900), Sylvain Poux (ORCID: 0000-0001-7299-6685), Catherine Rivoire (ORCID: 0000-0002-5979-8382), Shyamala Sundaram (ORCID: 0000-0003-4209-460X)

UniProt, EMBL-EBI: Alex Bateman (ORCID: 0000-0002-6982-4660), Aduragbemi Adesina (ORCID: 0000-0002-1029-4159) Emily Bowler-Barnett (ORCID: 0000-0003-4785-7231), David Carpentier (ORCID:0000-0002-8172-9121), Paul Denny (ORCID: 0000-0003-4659-6893), Alexandr Ignatchenko (ORCID: 0000-0002-6083-941X), Rizwan Ishtiaq (ORCID: 0000-0001-8041-7321), Antonia Lock (ORCID: 0000-0003-1179-5999), Yvonne Lussi (ORCID: 0000-0002-5753-0235), Michele Magrane (ORCID: 0000-0003-3544-996X), Maria J. Martin (ORCID: 0000-0001-5454-2815), Sandra Orchard (ORCID: 0000-0002-8878-3972), Pedro Raposo (ORCID: 0000-0001-6149-9456), Elena Speretta (ORCID: 0000-0003-1506-7438), Nidhi Tyagi (ORCID: 0000-0002-2065-9051), Nadya Urakova (ORCID: 0000-0003-3499-8158), Kate Warner (ORCID: 0000-0001-8705-181X), Conny Wing-Hen Yu (ORCID: 0000-0002-6478-5762)

WormBase: Raymond Lee (ORCID 0000-0002-8151-7479), Juancarlos Chan (ORCID:0000-0002-7259-8107), Stavros Diamantakis (ORCID:0000-0002-0273-3406), Mark Quinton-Tulloch (ORCID:0000-0001-5713-0736), Daniela Raciti (ORCID:0000-0002-4945-5837)

Xenbase: Malcolm Fisher (ORCID:0000-0003-1074-8103), Christina James-Zorn (ORCID:0000-0001-5495-4588), Virgilio Ponferrada (ORCID:0000-0002-8590-7183), Aaron Zorn (ORCID:0000-0003-3217-3590)

ZFIN: Doug Howe, Sridhar Ramachandran (ORCID:0000-0002-2246-3722), Leyla Ruzicka (ORCID:0000-0002-1009-339X), Monte Westerfield (ORCID:0000-0003-1187-7839)
