# Correction to ‘The Gene Ontology knowledgebase in 2026’

**DOI:** 10.1093/nar/gkag678

**Published:** 2026-06-30

This is a correction to: The Gene Ontology Consortium, The Gene Ontology knowledgebase in 2026, *Nucleic Acids Research*, Volume 54, Issue D1, 6 January 2026, Pages D1779–D1792, https://doi.org/10.1093/nar/gkaf1292

In the originally published version of this manuscript, Consortium author Daiqing Chen’s name was misspelled.

This error has been corrected.

